# High Expression of FAP in Colorectal Cancer Is Associated With Angiogenesis and Immunoregulation Processes

**DOI:** 10.3389/fonc.2020.00979

**Published:** 2020-07-08

**Authors:** Mairene Coto-Llerena, Caner Ercan, Venkatesh Kancherla, Stephanie Taha-Mehlitz, Serenella Eppenberger-Castori, Savas D. Soysal, Charlotte K. Y. Ng, Martin Bolli, Markus von Flüe, Guillaume P. Nicolas, Luigi M. Terracciano, Melpomeni Fani, Salvatore Piscuoglio

**Affiliations:** ^1^Institute of Pathology and Medical Genetics, University Hospital Basel, Basel, Switzerland; ^2^Visceral Surgery Research Laboratory, Department of Biomedicine, University of Basel, Basel, Switzerland; ^3^Department of Visceral Surgery, Clarunis University Centre for Gastrointestinal and Liver Diseases, St. Clara Hospital and University Hospital Basel, Basel, Switzerland; ^4^Department for BioMedical Research (DBMR), University of Bern, Bern, Switzerland; ^5^Division of Nuclear Medicine, University Hospital Basel, Basel, Switzerland; ^6^Division of Radiopharmaceutical Chemistry, University Hospital Basel, University of Basel, Basel, Switzerland

**Keywords:** colorectal cancer, cancer-associated fibroblast, immunohistochemistry, FAP, gene expression

## Abstract

Fibroblast activation protein α (FAP) plays an important role in tissue remodeling and helps tumor cells invade surrounding tissue. We sought to investigate FAP as a prognostic molecular marker in colorectal cancer (CRC) using immunohistochemical and transcriptomic data. *FAP* expression and clinicopathological information were obtained from The Cancer Genome Atlas data set. The association of *FAP* expression and tissue cellular heterogeneity landscape was explored using the xCell method. We evaluated FAP protein expression in a cohort of 92 CRCs and 19 non-tumoral tissues. We observed that *FAP* was upregulated in tumors both at the mRNA and protein levels, and its expression was associated with advanced stages, poor survival, and consensus molecular subtype 4. *FAP* expression was also associated with angiogenesis and collagen degradation. We observed an enrichment in immune-cell process–related genes associated with *FAP* overexpression. Colorectal cancers with high *FAP* expression display an inflamed phenotype enriched for macrophages and monocytes. Those tumors showed enrichment for regulatory T cell populations and depletion of T_H_1 and natural killer T cells, pointing to an immunosuppressive environment. Colorectal cancers with high levels of stromal FAP are associated with aggressive disease progression and survival. Our results suggest that FAP plays additional roles in tumor progression such as modulation of angiogenesis and immunoregulation in the tumor microenvironment.

## Introduction

Tumor-infiltrating immune cells as well as cancer-associated fibroblasts (CAFs) are important components of the tumor microenvironment. In human cancer, the tumor microenvironment has been suggested as a new component for the classification of malignant tumors including colorectal cancers (CRCs) (1–3). In particular, CAFs play important roles in modulating tumor development and prognosis via releasing proteolytic enzymes, growth factors, and immunomodulatory cytokines (4, 5).

Fibroblast activation protein α (FAP, also called seprase) is a prolyl-specific serine proteinase, highly upregulated in fibroblasts especially at sites of active tissue remodeling, including wound healing and fibrosis (6, 7). In CRC, previous studies reported the detection for FAP in more than 93% of the tumor. Among those, 30% showed high intensity for FAP staining (8). High FAP expression has been proposed as a biomarker for disease progression in metastatic CRCs (9). Similarly in rectal cancer, high FAP expression after preoperative chemoradiotherapy has been associated with poor prognosis (9). Given the scientific evidence, FAP has been considered as a candidate for targeted therapy in CRC. So far, diverse approaches, including FAP-targeting vaccines and immunotherapies, have been used in preclinical studies to deplete FAP-positive cells (10–12). Although these approaches have shown encouraging results in preclinical studies, those tested in clinical trials have shown limited efficacy, even in combination with chemotherapy (13–15). Furthermore, FAP-targeting radioligands have been used for *in vivo* imaging and targeted radionuclide therapy for a variety of cancers including CRC (16, 17).

Most of the functions described for FAP are associated with its enzymatic activity involved in tissue remodeling, which helps tumor cells invade the surrounding tissue, penetrate the blood vessel wall, and travel to form distant metastasis (18–21). Recent evidence suggested that FAP in CAFs could also play a critical role in regulating antitumor immune response by inducing tumor-promoting inflammation (22–24). This is particularly interesting because the majority of CRC patients are resistant to immunotherapies, especially to immune checkpoint blockades (25).

In our study, we sought to investigate FAP as a molecular marker in CRC using immunohistochemical and transcriptomic data. To investigate other potential roles of FAP in CRC, we explored its association with the clinicopathological characteristics of our in-house cohort. We further investigated its association at the mRNA level with molecular features, pathways and cell type populations in the tumor microenvironment using The Cancer Genome Atlas (TCGA) data set.

## Materials and Methods

### Patients and Specimen Characteristics

One hundred primary unselected, non-consecutive CRCs treated at the University Hospital Basel between the years 2006 and 2012 were included in this study. A tissue microarray (TMA) of these 100 tumors was constructed. Briefly, tissue cylinders with a diameter of 1 mm were punched from morphologically representative areas of each donor block and brought into one recipient paraffin block (30 × 25 mm) using the TMA GrandMaster® (TMA-GM; 3D-Histech Ltd.; Sysmex AG, Horgen, Switzerland) technology. Each punch was derived from the center of the tumor in an area with no necrosis so that each TMA spot consisted of more than 50% tumor cells. For 30 cases, non-malignant adjacent mucosa was selected from the same donor block. The study was performed in accordance with the Helsinki Declaration and approved by the ethics committee (Ethics Committee of Basel, EKBB, no. EKBB 361/12). Data were collected retrospectively in a non-stratified and non-matched manner including patient age, tumor diameter, location, pT/pN stage, grade, histologic subtype, vascular invasion, and clinical outcome. Intratumoral and peritumoral lymphocytic inflammation was evaluated using the original hematoxylin-eosin (H&E) slides of the resection specimens used as donor block. The tumor grade was categorized as low and high (≥50, <50% gland formation, respectively). The clinical outcome measure of interest was overall survival time.

### Immunohistochemistry

Immunohistochemistry (IHC) was performed using an anti-FAP antibody (Vitatex, Stony Brook, NY, USA; seprase/FAPα; dilution 1:100). Staining was performed on a Leica Bond III IHC staining system (Muttenz, Switzerland) using DAB as chromogen. Immunoreactivity was evaluated semiquantitatively as the proportion of positive staining in stromal cells in 10% increments, as well as the maximal staining intensity (0 = none, 1 = weak, 2 = intermediate, 3 = strong) by two experienced pathologists with expertise in gastrointestinal pathology (C.E. and L.M.T.). In terms of the percentage of FAP-positive cells, samples containing <10% of positive cells were classified as low, whereas samples containing at least 10% of positive cells were classified as high as suggested by Henry et al. (8). In terms of FAP staining intensity, samples with intensities 0 or 1 were considered low, whereas samples with intensities 2 or 3 were considered high (8). In addition, 20 cases positive for FAP on TMA were reevaluated using whole sections from formalin-fixed paraffin-embedded tissue to study FAP expression heterogeneity. FAP immunostaining was evaluated both in stroma adjacent to the invasive tumor front and within the tumor center.

### Tumor–Stromal Ratio

For all tumors, the tumor–stromal ratio on 4 μm H&E-stained tissue sections was calculated as described previously (26), and the stromal percentage was estimated per 10% intervals. Tumors were divided into stroma-high (>50%) and stroma-low (≤50%) groups according to their highest score.

### Assessment of Tumor Budding

Tumor budding was evaluated according to the International Tumor Budding Consensus Conference (ITBCC) method (27) and was defined according to ITBCC as single tumor cells or tumor cell clusters of up to four cells. Whole H&E-stained tissue sections of the tumors were used. One pathologist (C.E.) searched all tumor slides throughout at low magnification. Densest budding area at the invasive front (hot spot) was selected by visual estimation. Tumor buds in this area were counted at 20× magnification (field area, 0.785 mm^2^). Density of tumor buds was assigned into three grades: grade 1 (BD-1): 0–4 buds; grade 2 (BD-2): 5–9 buds; and grade 3 (BD-3): ≥10 buds.

### Microsatellite Instability

Immunohistochemical analyses of mismatch repair proteins were performed for expression of the four mismatch repair proteins MLH1, MSH2, MSH6, and PMS2 as previously described (28). Tissue samples with tumor cells lacking nuclear staining for at least one of these proteins were considered to have a positive microsatellite instability (MSI) screening status, hereafter referred to as MSI. Negative MSI screening status based on immunohistochemical staining is hereafter referred to as microsatellite-stable.

### Analysis of TCGA Data Set

FPKM gene-level expression data for TCGA colorectal carcinoma cohort (29) with 622 tumors and 51 non-tumoral tissues, defined as “solid tissue normal,” were obtained from TCGA Genomics Data Commons harmonized data portal using *TCGAbiolinks* R package (30). The expression of *FAP* was compared between tumors and normal tissues using the Student *t*-test. Tumor samples were classified into FAP-high and FAP-low groups based on the threshold of mean + 3 standard deviations of normal tissues. Clinical information was obtained from the Human Protein Atlas (Pathology Atlas) (31) CRC project for 596 TCGA CRCs.

Raw read counts of the TCGA CRC Project downloaded using *TCGAbiolinks* package (30) were used for differential expression analysis using the *edgeR* package (32). Genes with low expression (<1 log-counts per million in ≥50 samples) were filtered out. Normalization was performed using the “TMM” (weighted trimmed mean) method (33), and differential expression was assessed using the quasi-likelihood *F* test. Genes with log-fold change >2 and false discovery rate (FDR) <0.05 were considered differentially expressed. Pathway enrichment analysis of the upregulated genes from the differential analysis between the *FAP*-high and *FAP*-low groups was performed using clusterProfiler package (34), which supports Gene Ontology, KEGG, and Reactome Pathways. Significantly enriched pathways were selected based on FDR <0.05. Gene set enrichment analysis (GSEA) of all analyzed genes ranked based on signed *p* value according to the direction of the log-fold change was performed using the *fgsea* package (35). Gene Ontology gene sets from MSigDB (36) were used to identify significantly upregulated/downregulated pathways. Molecular subtyping was performed using *CMScaller* package (37), and the 622 TCGA CRCs were classified into 97 CMS1, 170 CMS2, 95 CMS3, 195 CMS4 subtypes, and 65 unclassified. Cell type enrichment analysis was performed with FPKM gene expression data using *xCell* gene signatures-based method for cell types (38).

### Statistical Analysis

Statistical comparisons between categorical variables were performed using χ^2^ test or Fisher exact test where appropriate. Statistical comparisons between numeric variables were performed using *t*-test, Mann–Whitney *U*-test, or paired Wilcoxon test. Survival analysis was performed using the Kaplan–Meier method and log-rank test. For the TCGA cohort, stratification of *FAP* expression for overall survival analysis was performed using the *maxstat* R package (39). Univariate Cox regression analyses were performed to investigate the association between overall survival and clinical variables. Variables significant in univariate Cox regression analyses were included in multivariate Cox regression analysis. All tests were two-sided, and *p* < 0.05 was considered statistically significant. Statistical power of statistical tests was estimated by 100 iterations of bootstrapping. Statistical analysis was carried out with Prism (v7.0; San Diego, CA, USA) and R (v.3.6.1; R Foundation for Statistical Computing, Vienna, Austria. http://www.R-project.org/).

## Results

### Expression of FAP in Colorectal Cancers

**Figure 1:**
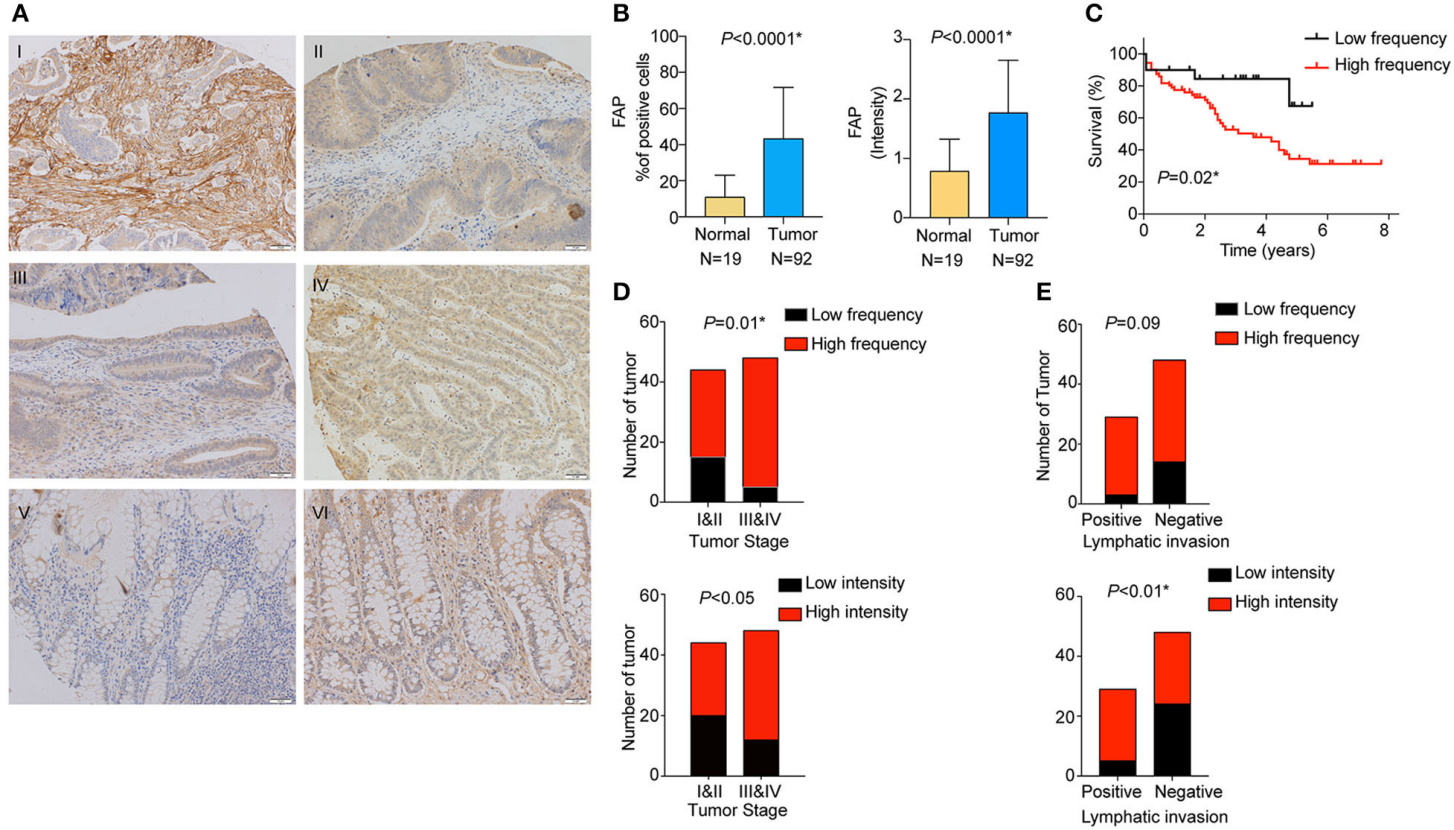
FAP protein is overexpressed in CRC and is associated with worse overall survival. **(A)** Representative examples of FAP expression by immunohistochemistry. FAP expression in (I) high percentage of tumor-associated stromal cells (*100%*); (II) tumor-associated stroma with low number of positive cells *(5%*); (III) normal tissue showing negative staining for FAP; (IV) FAP high-intensity staining in tumor-associated stromal cells (*high*); *(V)* FAP low-intensity staining in tumor-associated stromal cells (*low*); and (VI) normal tissue showing FAP high-intensity staining in stroma cells. Scale bar: 50 μm. **(B)** Percentage of cells showing positive FAP staining in normal stroma and tumor-associated stroma (above). Scoring of FAP staining based on intensity in normal and tumor-associated stroma (below). **(C)** Overall survival analysis (Kaplan–Meier) of CRC patients from the TMA cohort after stratification for high and low percentage of FAP-positive cells. Number of CRCs with high and low percentages of FAP-positive cells (above), as well as high and low FAP intensity (below) in **(D)** early and advanced tumor stages, and **(E)** positive and negative lymphatic invasion. Statistical analyses were performed using Fisher exact test for categorical variables, Mann–Whitney *U*-test for numeric variables and log-rank (Mantel–Cox) test for survival analysis. All tests were two-sided, and *p* < 0.05 was considered statistically significant. Data in **(B)** are represented as mean ± SD. *Statistical power >70%.

We analyzed the expression of FAP at the protein level in stromal cells by staining and scoring a TMA containing 100 CRC samples and 30 non-tumoral adjacent tissues using IHC (Figure 1A). After excluding samples for which the tissue core was absent or had poor staining quality, 92 CRCs and 19 non-tumoral colon samples were available for analysis. We observed a significantly higher percentage of FAP-positive stromal cells in tumors compared to non-tumoral tissues (*p* < 0.0001; Figure 1B). FAP expression was detected in 91% (84/92) of the tumors. High frequency (at least 10%) of FAP-positive cells was found in 78% (72/92) of CRCs, whereas high FAP intensity was observed in 66% (61/92, Figure 1 and Supplementary Figure 1A). Sixty-three percent (58/92) had high frequency of FAP-positive cells with elevated intensity (2 or 3). Although FAP staining was detectable in 79% (15/19) of the non-tumoral tissue samples, both the number of positive cells and the intensity were significantly lower compared to tumoral tissue (both *p* < 0.0001; Figure 1B). Similar results were observed when we considered the 19 matched pairs of CRCs and non-tumoral colon samples (*p* = 0.005 for frequency and *p* = 0.01 for intensity; Supplementary Figure 1B). Of the 15 non-tumoral tissue samples with detectable FAP expression, only a single case showed a high percentage (≥10%) of FAP-positive (intensity 2) cells.

We then investigated the association between FAP expression (both frequency and intensity) and the clinicopathological characteristics of our cohort (Table 1). We found that the frequency of FAP-positive cells and that of cells with high FAP intensity were significantly more frequently found in CRCs with advanced stages (*p* = 0.01 and *p* < 0.05, respectively, Figure 1D). Similarly, high FAP intensity was also associated with tumors showing high tumor grade and lymphovascular invasion (*p* < 0.05 and *p* = 0.03, respectively, Table 1). The lymphatic invasion was also associated with a high frequency of FAP-positive cells (*p* = 0.09, Figure 1E and Table 1) and a high FAP intensity (*p* < 0.01, Figure 1E and Table 1). We further observed a stepwise increase in tumor budding according to the percentage of FAP-positive cells (*p* = 0.009, Supplementary Figure 1C). No significant association was found with age, sex, tumor location, presence of MSI, stroma-to-tumor ratio, or venous invasion (Table 1).

**Table 1:** Association between FAP protein expression and clinicopathological features in the TMA cohort.

**Clinical features**	**Frequency**			**Intensity**		
	**High FAP expression**	**Low FAP expression**	***p***	**High FAP expression**	**Low FAP expression**	***p***
**Age (years)**						
<59	9 (82%)	2 (18%)	0.64	7 (64%)	4 (36%)	0.11
60–69	16 (73%)	6 (27%)		11 (50%)	11 (50%)	
70–79	24 (86%)	4 (14%)		23 (82%)	5 (18%)	
>80	23 (74%)	8 (26%)		19 (61%)	12 (39%)	
**Sex**						
Male	42 (78%)	12 (22%)	1	34 (63%)	20 (37%)	0.66
Female	30 (79%)	8 (21%)		26 (68%)	12 (32%)	
**Tumor location**						
Cecum	14 (82%)	3 (18%)	0.96	13 (76%)	4 (24%)	0.73
Ascending colon	12 (80%)	3 (20%)		8 (53%)	7 (47%)	
Transverse colon	4 (80%)	1 (20%)		4 (80%)	1 (20%)	
Descending colon	6 (67%)	3 (33%)		5 (56%)	4 (44%)	
Sigmoid colon	20 (77%)	6 (23%)		16 (62%)	10 (38%)	
Rectum	16 (80%)	4 (20%)		13 (65%)	7 (35%)	
**Stage**						
I	10 (59%)	7 (41%)	0.03	11 (65%)	6 (35%)	0.14
II	19 (70%)	8 (30%)		13 (48%)	14 (52%)	
III	25 (93%)	2 (7%)		20 (74%)	7 (26%)	
IV	18 (86%)	3 (14%)		16 (76%)	5 (24%)	
**Grade**						
Low	51 (75%)	17 (25%)	0.26	40 (59%)	28 (41%)	<0.05
High	21 (88%)	3 (12%)		20 (83%)	4 (17%)	
Tumor: stroma^*^						
Stroma low	55 (77%)	16 (23%)	1	46 (65%)	25 (35%)	1
Stroma high	17 (68%)	4 (32%)		14 (66%)	7 (34%)	
Microsatellite instability^*^						
MSI	9 (90%)	1 (10%)	0.45	9 (90%)	1 (10%)	0.09
MSS	62 (77%)	19 (23%)		50 (62%)	31 (38%)	
**Lymphatic invasion**						
Positive	26 (90%)	3 (10%)	0.09	24 (83%)	5 (17%)	<0.01^**^
Negative	34 (71%)	14 (29%)		24 (50%)	24 (50%)	
Venous invasion^*^						
Positive	16 (76%)	5 (24%)	1	13 (61%)	8 (38%)	1
Negative	44 (76%)	14 (24%)		35 (60%)	23 (40%)	
Lymphovascular invasion^*^						
Positive	32 (84%)	6 (16%)	0.2	29 (76%)	9 (24%)	0.03
Negative	33 (72%)	13 (28%)		24 (52%)	22 (48%)	

*All 2 × 2 contingency tables were analyzed with Fisher exact tests. All others by χ^2^ test*.

* *Patients with data not available, unknown, and discrepancies*.

** *Statistical power estimated by bootstrapping*.

Furthermore, we determined whether there was an association between the frequency of FAP-positive cells and overall survival. High frequency of FAP-positive cells, but not FAP intensity, was associated with worse overall survival (*p* = 0.02; Figure 1C and Supplementary Figure 1D). Similarly, univariate and multivariate Cox regression analyses showed that FAP expression is an independent predictor of overall survival (Supplementary Table 1).

**Figure 2:**
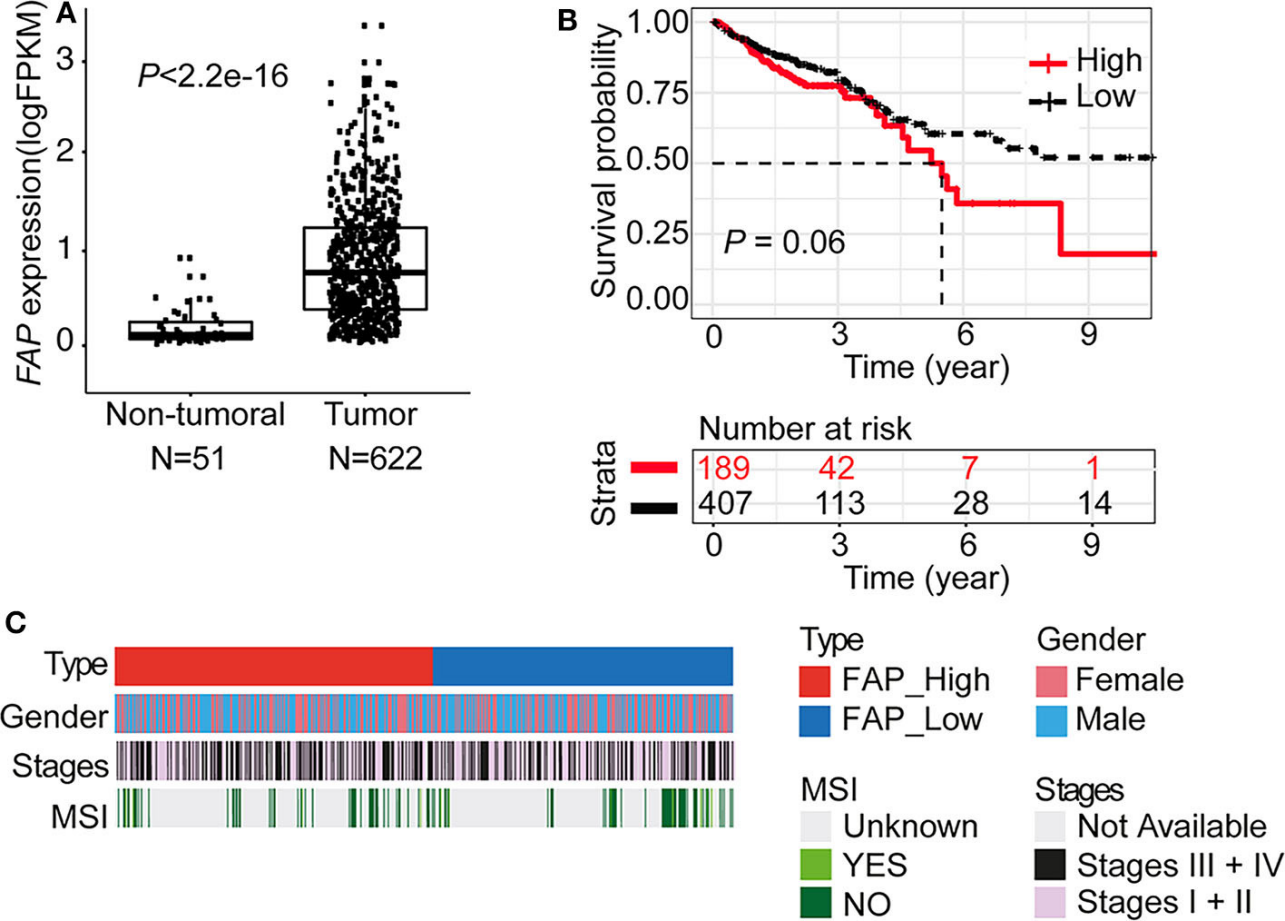
*FAP* is upregulated in colorectal tumors. **(A)** *FAP* expression level in tumors (*n* = 622) compared to non-tumoral tissue (*n* = 51) in the TCGA data set. **(B)** Overall survival analysis (Kaplan–Meier) of CRC patients from the TCGA data set stratified by *FAP* expression (see also Supplementary Figure 2). **(C)** Colorectal cancer patient characteristics grouped by *FAP* expression. Statistical comparisons of clinical and molecular parameters between tumors with high vs. low *FAP* expression are shown in Table 2. Statistical comparisons were performed Mann–Whitney *U*-test in **(A)** and by log-rank test in **(B)**.

To cross-validate our results, we retrieved the gene expression data of 622 CRC cases from TCGA (29). In agreement with the data obtained by IHC on the TMA (Figure 1B), tumor samples expressed significantly higher levels of *FAP* compared to normal tissues (*p* < 2.2e-16; Figure 2A). Association of *FAP* expression with clinicopathological parameters demonstrated that *FAP* overexpression was associated with more advanced tumor stage (*p* = 0.02, Table 2 and Figure 2C). In terms of outcome, we observed a trend toward worse overall survival in patients with tumors with high *FAP* expression (*p* = 0.06; Figure 2B and Supplementary Figure 2). Univariate analysis found tumor stages and tumor location as predictors of overall survival, whereas *FAP* expression showed a trend to it (*p* = 0.06; Supplementary Table 2).

**Table 2:** Association between *FAP* mRNA expression and clinicopathological features in the TCGA cohort.

**Clinical features**		**Low *FAP* expression**	**High *FAP* expression**	***p***
		**n (%)**	**n (%)**	
Gender (*n* = 591)	Female (*n* = 271)	133 (49.1%)	138 (50.9%)	0.81
	Male (*n* = 320)	154 (48.1%)	166 (51.9%)	
AJCC stages (*n* = 571)^*^	Stage I + II (*n* = 316)	167 (52.8%)	149 (47.2%)	0.02
	Stage III + IV (*n* = 255)	111 (43.5%)	144 (56.5%)	
Microsatellite instability (*n* = 115)^*^	MSI (*n* = 11)	7 (63.6%)	4 (36.4%)	0.42
	MSS (*n* = 104)	53 (51.0%)	51 (49.0%)	
CRC subtyping (*n* = 556)^*^	CMS1 (*n* = 97)	36 (37.1%)	61 (62.9%)	<0.001^**^
	CMS2 (*n* = 170)	129 (75.9%)	41 (24.1%)	
	CMS3 (*n* = 94)	66 (70.2%)	28 (29.8%)	
	CMS4 (*n* = 195)	17 (8.7%)	178 (91.3%)	
CRC location (*n* = 597)^*^	Cecum (*n* = *n* = 106)	56 (52.8%)	50 (47.2%)	0.4
	Ascending colon (*n* = 86)	31 (36.0%)	55 (64.0%)	
	Descending colon (*n* = 20)	9 (45.0%)	11 (55.0%)	
	Transverse colon (*n* = 38)	20 (52.6%)	18 (47.4%)	
	Sigmoid colon (*n* = 155)	75 (48.4%)	80 (51.6%)	
	Hepatic flexure (*n* = 26)	12 (46.2%)	14 (53.8%)	
	Splenic flexure (*n* = 7)	4 (57.0%)	3 (43.0%)	
	Rectosigmoid junction (*n* = 49)	24 (49.0%)	25 (51.0%)	
	Rectum (*n* = 110)	60 (55.0%)	50 (45.0%)	

* *Patients with data not available, unknown, and discrepancies*.

** *Statistical power >70% (estimated by bootstrapping)*.

*All 2 × 2 contingency tables were analyzed with Fisher exact tests. All others by χ^2^ test*.

Taken together, our results suggest that FAP expression may be prognostic in CRC.

### Transcriptomic Analysis of *FAP* Expression in Colorectal Tumors

**Figure 3:**
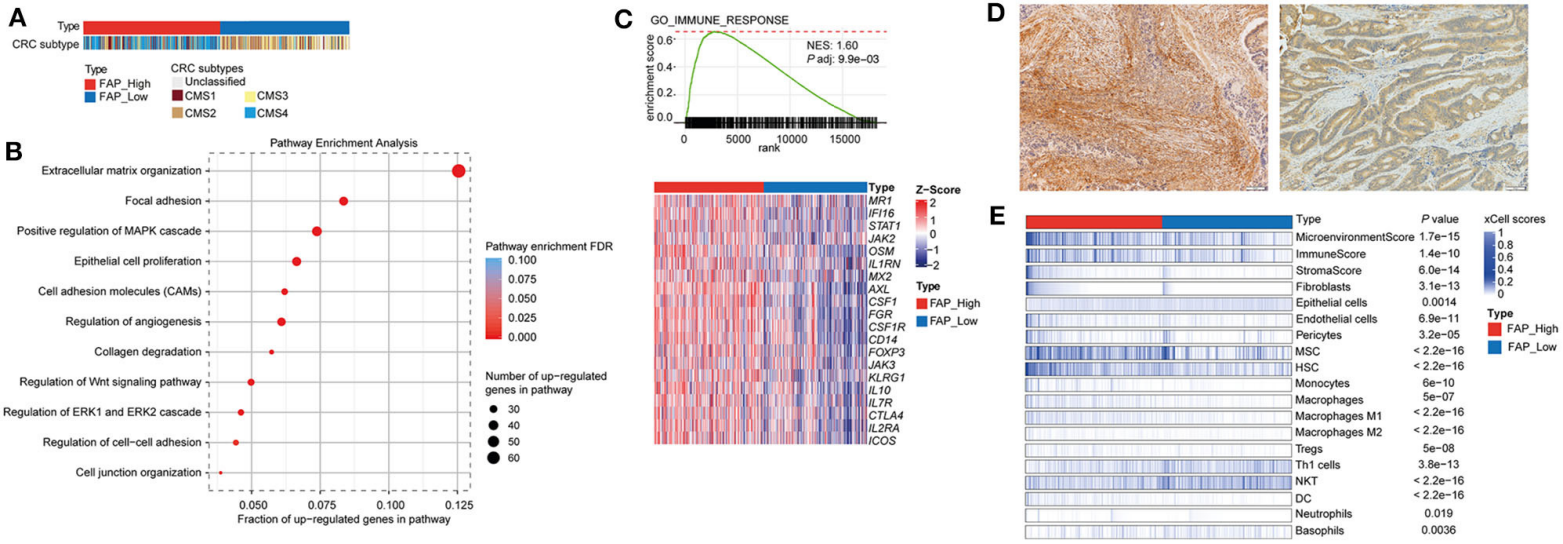
*FAP* is associated with pathways involved in tumor growth, invasion, and immunosuppressive tumor microenvironment. **(A)** Colorectal cancers grouped by *FAP* expression. Statistical comparison of molecular subtypes between tumors with high vs. low *FAP* expression are shown in Table 2. **(B)** Figure shows selected significantly enriched pathways from pathway enrichment analysis of the upregulated genes from a differential expression analysis between *FAP*-high vs. *FAP*-low. The size of the dots indicates the number of upregulated genes in each pathway. The color of the dot indicates FDR, and *x* axis represents the fraction of upregulated genes in the pathway. **(C)** Gene set enrichment analysis plots of GO immune response, where *x* axis shows ranked list of genes (ranked by the *p*-values signed according to the direction of the differential expression analysis between *FAP*-high and *FAP*-low CRCs), and the vertical bars on the *x* axis show the genes that belong to gene set. The *y* axis shows the enrichment score of the gene set. Heatmaps below show selected genes in the GO process. **(D)** Representative pictures showing the enrichment of fibroblast on high *FAP* expression (left) and low number of fibroblasts on low *FAP* expression (right). **(E)** Heatmap shows the enrichment of immune and stromal cell types in the TCGA CRC cohort, as defined by the xCell method. Samples were stratified into *FAP*-high and *FAP*-low groups and then sorted based on their xCell fibroblasts scores. Cell types that showed statistically significant difference between *FAP*-high and *FAP*-low groups are shown. Statistical comparisons were performed using Mann–Whitney *U*-tests.

To further understand the possible role of *FAP* in CRC, we analyzed the transcriptomic data from the TCGA data set (*n* = 622). We investigated the association of *FAP* expression with the CRC molecular subtypes, and we observed a statistically significant association with tumors classified as CMS1 and CMS4 (*p* = 0.02 and *p* < 0.001; Figure 3A and Table 2). CMS1 and CMS4 have been reported to be associated with an upregulation of immune response genes and epithelial-to-mesenchymal transition, respectively (40). In particular, the association with CMS4 suggests a more aggressive origin of these tumors characterized by *FAP* overexpression.

Then, we performed a differential expression analysis between CRCs with high vs. low *FAP* expression. The differential expression analysis revealed 655 up- and 9 downregulated genes. Consistent with previous reports (41, 42), we found that the upregulated genes were enriched in functions related to collagen degradation, extracellular organization, regulation of cell-to-cell adhesion, and cell junction organization (Figure 3B). Additionally, pathways involved in epithelial cell proliferation, invasion, and immune surveillance such as regulation of Wnt signaling, ERK1, and ERK2 cascade and angiogenesis were also associated with *FAP* upregulation (Figure 3B). We further performed a GSEA by ranking all expressed genes based on the signed *p*-value from the differential expression analysis according to the direction of the log-fold change (Supplementary Figure 3). We observed that *FAP* overexpression was associated with processes related to epithelial-to-mesenchymal transition, angiogenesis, tissue remodeling, epithelial cell proliferation, and immune response (Supplementary Figures 3, 4).

Because we observed that *FAP* overexpression was associated with immune-related processes (Figure 3C and Supplementary Figure 3), we sought to explore which immune components may be involved. We observed that CRCs with high *FAP* expression showed high expression of genes such as *FOXP3, CTL4, ICOS*, and *KLRG1* (Figure 3C), which are usually expressed in immune cell populations such as regulatory T cells (Tregs) or in populations showing an immunosuppressive phenotype. To evaluate whether *FAP* overexpression was associated with the enrichment of specific immune cell populations, we used xCell, a method to perform cell type enrichment analysis from gene expression data for 64 immune and stromal cell types, on the TCGA CRC cohort. Overall, we found that tumors with high *FAP* expression were enriched for both immune and stromal cell types compared to tumors with low *FAP* expression (Figure 3D and Supplementary Figure 5). Populations such as endothelial cells and fibroblasts were found to be more abundant in *FAP*-overexpressing CRC (*p* = 3.1e-13 and *p* = 6.9e-11, respectively; Figures 3D,E). Similarly, macrophages, monocytes, and Tregs were also enriched in *FAP*-overexpressing samples. Interestingly, *FAP*-overexpressing CRCs were depleted for populations associated with antitumoral responses such as T_H_1 cells and natural killer T (NKT) cells. No significant differences were found regarding CD8 T cells, T_H_2 cells, or B cells.

Taken together, our results suggest that FAP may contribute to the poor prognosis of CRC by modulating the tumor microenvironment not only by driving angiogenesis but also by promoting a more protumorigenic environment.

### FAP Distribution in the Tumor Center and Invasive Tumor Front

**Figure 4:**
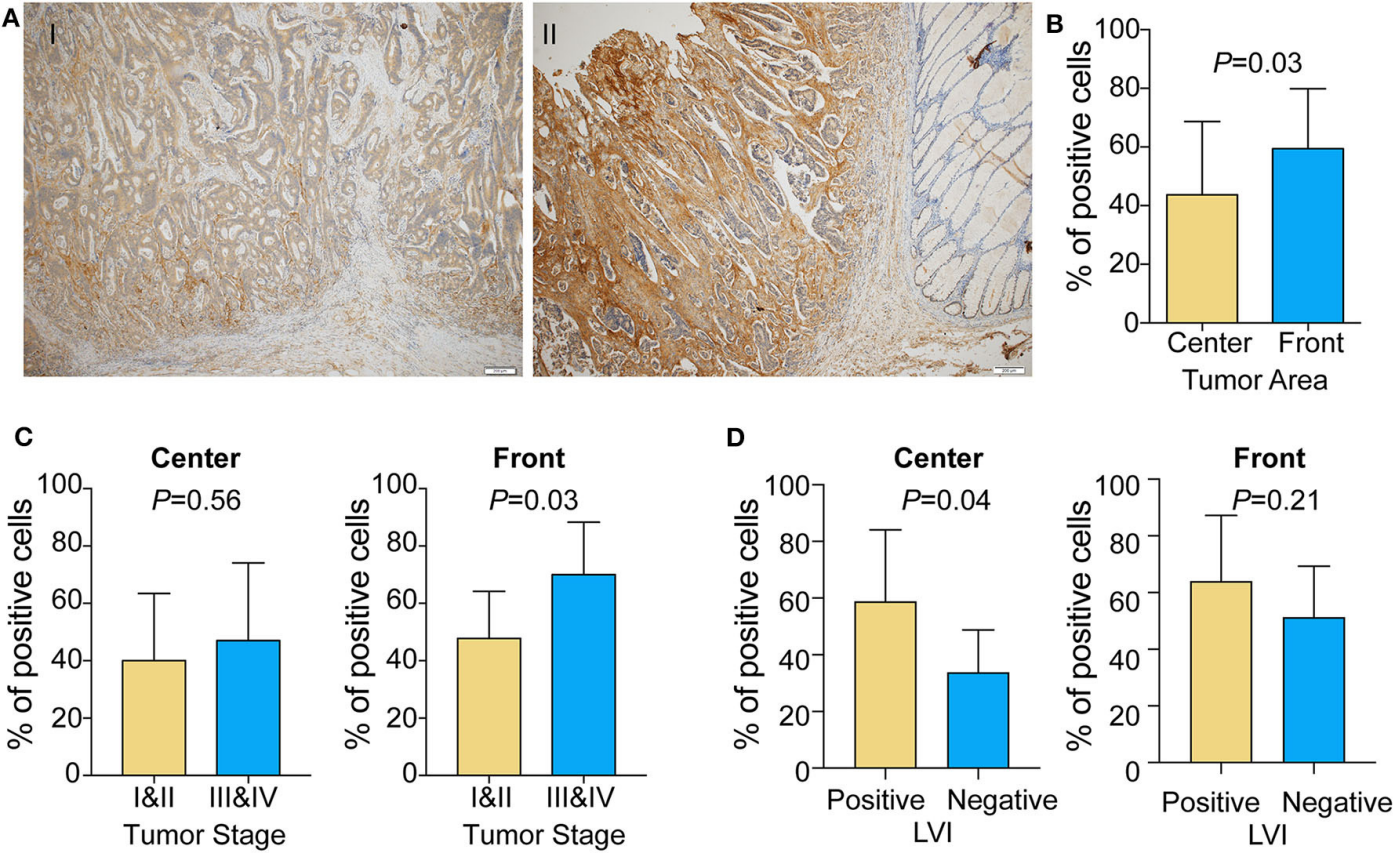
FAP expression is enriched in the tumor front area in colorectal tumor. **(A)** Representative micrographs of FAP distribution in the tumor center and at the tumor front area. (I) FAP expression is higher at the tumor front compared to the tumor center; (II) FAP expression does not show significant difference between tumor center and tumor front, whereas neighbor non-neoplastic colonic mucosal stroma is completely negative. Scale bar: 200 μm. **(B)** Percentage of cells showing FAP-positive staining in the tumor front and tumor center areas (*n* = 19). **(C)** Percentage of cells showing FAP-positive staining in tumor center (left) and front (right) in early and advanced tumor stage. **(D)** Percentage of cells showing FAP-positive staining in tumor center (left) and front (right) with lymphovascular invasion. Statistical analyses were performed using Fisher exact tests for categorical variables and Mann–Whitney *U*-tests for numeric variables. All tests were two-sided, and *p* < 0.05 was considered statistically significant. Data in **(B–D)** are represented as mean ± SD.

Given that the transcriptomic analysis suggests that FAP may promote a protumorigenic environment, we investigated whether the localization of FAP in the tumor could be associated with its role in CRC invasion. We took advantage of 20 CRCs that showed positive FAP staining in the TMA and stained whole tissue sections to define FAP heterogeneity. Using whole sections, we were able to visualize the tumor center and the invasive front in 95% (19/20) of the samples. FAP staining was visible in both tumor center and tumor front in all 19 cases (Figure 4A). Compared to the tumor center, FAP-positive cells were more frequently found at the invasive front (*p* = 0.03; Figure 4B). The high frequency of FAP-positive cells at the invasive front was associated with advanced tumor stage (*p* = 0.03; Figure 4C; right), but no significant association was found between the frequency of FAP-positive cells in the tumor center and tumor stage (Figure 4C; left). The presence of lymphovascular invasion was significantly associated with high frequency of FAP-positive cells in tumor center (Figure 4D, *p* = 0.04) but not in tumor front.

These results are in line with the results we obtained by TMA, underlying the important role of FAP in tumor invasion.

## Discussion

FAP has been shown to be overexpressed in tumor-associated stromal cells in epithelial tumors (43) and its presence has been associated with worse prognosis. Moreover, tumors showing upregulation of FAP present a high level of microvessel density (44), which is also a marker for poor prognosis in several epithelial cancers (45–47). *In vivo* studies have shown that FAP overexpression in breast and colonic xenograft models leads to more rapid development of subcutaneous tumors and enhanced tumor growth (44). By contrast, inhibition of FAP activity in colorectal xenograft models results in tumor growth attenuation (48). Together, there is substantial evidence supporting the role of FAP in tumor proliferation and metastasis (49). Moreover, little is known about the molecular role of FAP in CRC and its potential in modulating the tumor microenvironment. This is particularly important because immune checkpoint inhibitors have demonstrated little or no clinical activity in the majority of patients with metastatic CRC (50).

In the present study, we investigated the expression of FAP both at the RNA and protein levels in two independent cohorts of CRC and its association with clinicopathological features. Similarly to previous reports evaluating FAP expression using IHC (8, 9, 51, 52), FAP was found upregulated in tumors compared to non-tumoral tissues and was associated with poor survival in both cohorts. In line with the poor prognosis, we found that high frequency of FAP-positive cells and high FAP intensity at the protein and RNA levels were associated with advanced stages. Although our results are discrepant from previous works showing FAP expression was also elevated in early-stage CRC (8, 51), we also found that FAP expression was associated with lymphatic invasion and tumor budding. Lymphatic invasion has been used to estimate the aggressiveness of colorectal tumors (53), whereas tumor budding is a surrogate for epithelial-to-mesenchymal transition and is associated with poor prognosis (54). The findings related to pathological features are thus in agreement with the association with advanced stages. Taken together, the association of FAP expression on the mRNA and protein levels with multiple clinicopathological features known to be associated with poor prognosis supports our finding that FAP is also associated with tumor aggressiveness.

Most studies on FAP have focused on its potential value as a prognostic marker in epithelial cancers, but little is known on how and why FAP may be prognostic. We, therefore, analyzed the transcriptomic data from 622 CRCs in the TCGA. We found that the majority of tumors with high *FAP* expression were classified as CMS1 and CMS4 of the consensus molecular subtypes. CMS4 tumors have been reported to overexpress proteins implicated in stromal invasion, mesenchymal activation, and complement pathways. The enrichment for CMS4 tumors among tumors with high *FAP* expression suggests a more invasive tumor phenotype, which agrees with our findings from the TMA. In fact, our analysis of the TCGA transcriptomic data also showed that CRCs with *FAP* overexpression were enriched for stromal cell types, in particular for fibroblasts and mesenchymal stem cells. Furthermore, most of the pathways associated with *FAP* overexpression were related to extracellular matrix, junction remodeling, and collagen degradation, all of which have previously been associated with FAP expression. We further found that epithelial-to-mesenchymal transition, angiogenesis, and epithelial cell proliferation were also enriched in *FAP*-overexpressing CRCs. Our results are in line with previous studies that have demonstrated the involvement of FAP in epithelial-to-mesenchymal transition, angiogenesis, and in tumorigenesis (44, 49, 55). Additionally, our IHC analysis showed that the frequency of FAP-positive cells was associated with tumor budding score, a very well-known pathological marker associated with epithelial-to-mesenchymal transition and tumor invasion. Overall, the findings from our analysis of the TCGA are further supported by the observation that FAP was preferentially found at the tumor invasive front, in agreement with Sandberg et al. (56), suggesting a role in tissue invasion and metastasis.

In addition to CMS4, we also found that *FAP*-overexpressing CRCs were enriched in the subtype CMS1. While the most widely described function of FAP on CAFs in CRC was extracellular matrix remodeling (57), new evidence suggests that FAP on CAFs also has critical roles in regulating antitumor immune response by inducing tumor-promoting inflammation (58). Indeed, the CMS1 subtype displays upregulation of proteins involved in immune response pathways (40). We also found that *FAP* overexpression is associated with an upregulation of genes involved in immune cell response, suggesting that FAP may promote an inflamed environment. Recent reports have proposed that FAP expression may be associated with resistance to immune therapies (22, 59). Using the xCell algorithm, we found that populations such as T_H_1 and NKT cells were suppressed, whereas Tregs were enriched in *FAP*-overexpressing CRCs, suggesting an immunosuppressive environment in these tumors. Chen et al. (23) reported that FAP expression promotes immunosuppression in a CRC tumor model via the upregulation of CCL2. CCL2, a member of the C-C chemokine family, regulates the recruitment of myeloid cells, mostly macrophages and monocytes (60), into inflamed sites to promote tumor growth (61, 62). In breast cancer, Costa et al. (63) showed that CAF with high expression of FAP was associated with increased CD25^+^FOXP3^+^ T lymphocytes via the modulation of B7H3, DPP4, and CD73. Accordingly, we also observed that macrophage and monocyte populations are enriched in *FAP*-overexpressing CRCs.

Although the statistical power of our study is limited by the relatively small sample size of the TMA cohort, our study is still one of the largest cohorts evaluating FAP expression in CRCs. Our results support the well-known role of FAP in promoting tumor growth and invasion. Based on our results, we speculate that one of the mechanisms by which FAP promotes tumorigenesis is linked to its ability to recruit endothelial cells and to induce angiogenesis, together with its enzymatic activity. Moreover, FAP will orchestrate a broad panel of other cells to push microenvironment toward an immunosuppressive environment, thus providing a niche for a more aggressive CRC phenotype.

## Data Availability Statement

Publicly available datasets were analyzed in this study. This data can be found here: TCGA data portal (https://portal.gdc.cancer.gov/).

## Ethics Statement

The studies involving human participants were reviewed and approved by Ethics Committee of Basel, EKBB, number EKBB 361/12. The patients/participants provided their written informed consent to participate in this study.

## Author Contributions

MC-L, LT, MF, and SP: conceived the study. MC-L and SP: supervised the study. VK: performed bioinformatic analysis. CE and LT: performed the histologic review and immunohistochemical evaluation. MC-L, CE, ST-M, and SP: analyzed the results. SS, MB, MvF, GN, and MF: carefully discussed the results. MC-L, CE, and VK: wrote the manuscript that was revised by CN and SP. All authors edited and approved the final draft of the manuscript and are accountable for all aspects of the work.

## Conflict of Interest

The authors declare that the research was conducted in the absence of any commercial or financial relationships that could be construed as a potential conflict of interest.

## Supplementary Material

Supplementary Figure 1 **(A)** Table displays the distribution of frequency of FAP-positive cells and FAP intensity in the TMA cohort. **(B)** Frequency (left) and intensity (right) of FAP-positive cells in 19 non-malignant tissues and paired malignant tissues. **(C)** Percentage of cells showing FAP-positive staining (left) or intensity (right) associated with tumor budding score. **(D)** Overall survival analysis (Kaplan–Meier) of CRC patients from the TMA cohort after stratification (below) for tumors with high FAP intensity vs tumors with low FAP intensity and (above) for tumors with high frequency of FAP-positive cells and/or high FAP intensity vs tumors with low frequency of FAP-positive cells and low FAP intensity. Data in **(B,C)** are represented as mean ± SD Statistical analyses were performed using Fisher exact tests for categorical variables in **(B)**, ANOVA in **(C)** and log-rank (Mantel–Cox) tests for survival analysis in **(D)**. All tests were two-sided, and *p* < 0.05 was considered statistically significant. ^*^Statistical power >70%.

Supplementary Figure 2 The plot shows the optimal cutpoint for the stratification of TCGA CRC cohort into two groups, *FAP*-high and *FAP*-low, for overall survival analysis.

Supplementary Figure 3 **(A)** Gene Set Enrichment Analysis plot shows selected significantly enriched Gene Ontology gene sets from MSigDB database in *FAP*-high tumors in the TCGA CRC cohort. **(B)** Gene Set Enrichment Analysis plots of Gene Ontology molecular functions, where x-axis shows ranked list of genes (ranked by the *p*-values signed according to the direction of the differential expression analysis between *FAP*-high and *FAP*-low CRCs) and the vertical bars on the x-axis show the genes that belong to gene set. The y-axis shows the enrichment score of the gene set. Heatmaps below show selected genes in each GO process. Pearson correlation coefficients of the expression of the genes with *FAP* expression are shown to the right. NES: normalized enrichment score. P adj, *p*-value adjusted for multiple testing (i.e., FDR).

Supplementary Figure 4 Correlation of *FAP* expression with **(A)** immune markers; **(B)** epithelial to mesenchymal transition (EMT) markers and **(C)** angiogenesis markers. Statistical analyses were performed using Pearson correlation tests.

Supplementary Figure 5 Boxplots show the enrichment scores of each cell type between *FAP*-high and *FAP*-low groups in the TCGA CRC cohort, as defined by xCell. p-values were calculated using t-tests.

Supplementary Table 1 Univariate and multivariate Cox regression analyses for the effect of FAP protein expression and clinicopathological parameters on overall survival in the TMA cohort.

Supplementary Table 2 Univariate and multivariate Cox regression analyses for the effect of *FAP* mRNA expression and clinicopathological parameters on overall survival in the TCGA cohort.
