# Supplementary figures and images for: High Expression of FAP in Colorectal Cancer Is Associated With Angiogenesis and Immunoregulation Processes
Source: Front Oncol. 2020 Jul 8;10:979. doi: 10.3389/fonc.2020.00979 (PMC7362758; doi:10.3389/fonc.2020.00979)

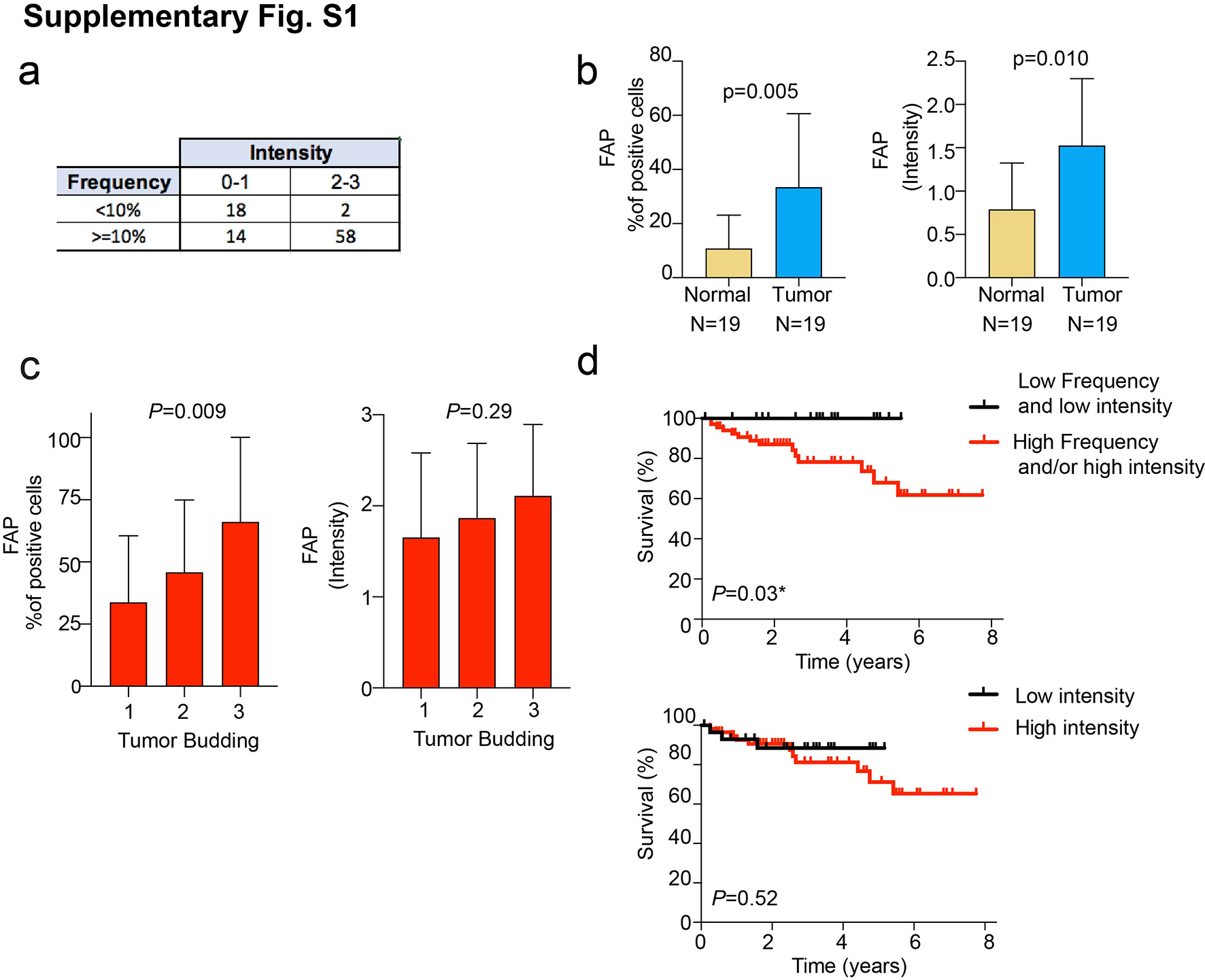

Supplement: Supplementary Figure 1 — (A) Table displays the distribution of frequency of FAP-positive cells and FAP intensity in the TMA cohort. (B) Frequency (left) and intensity (right) of FAP-positive cells in 19 non-malignant tissues and paired malignant tissues. (C) Percentage of cells showing FAP-positive staining (left) or intensity (right) associated with tumor budding score. (D) Overall survival analysis (Kaplan–Meier) of CRC patients from the TMA cohort after stratification (below) for tumors with high FAP intensity vs tumors with low FAP intensity and (above) for tumors with high frequency of FAP-positive cells and/or high FAP intensity vs tumors with low frequency of FAP-positive cells and low FAP intensity. Data in (B,C) are represented as mean ± SD Statistical analyses were performed using Fisher exact tests for categorical variables in (B), ANOVA in (C) and log-rank (Mantel–Cox) tests for survival analysis in (D). All tests were two-sided, and p < 0.05 was considered statistically significant. *Statistical power >70%. [file Image_1.JPEG]

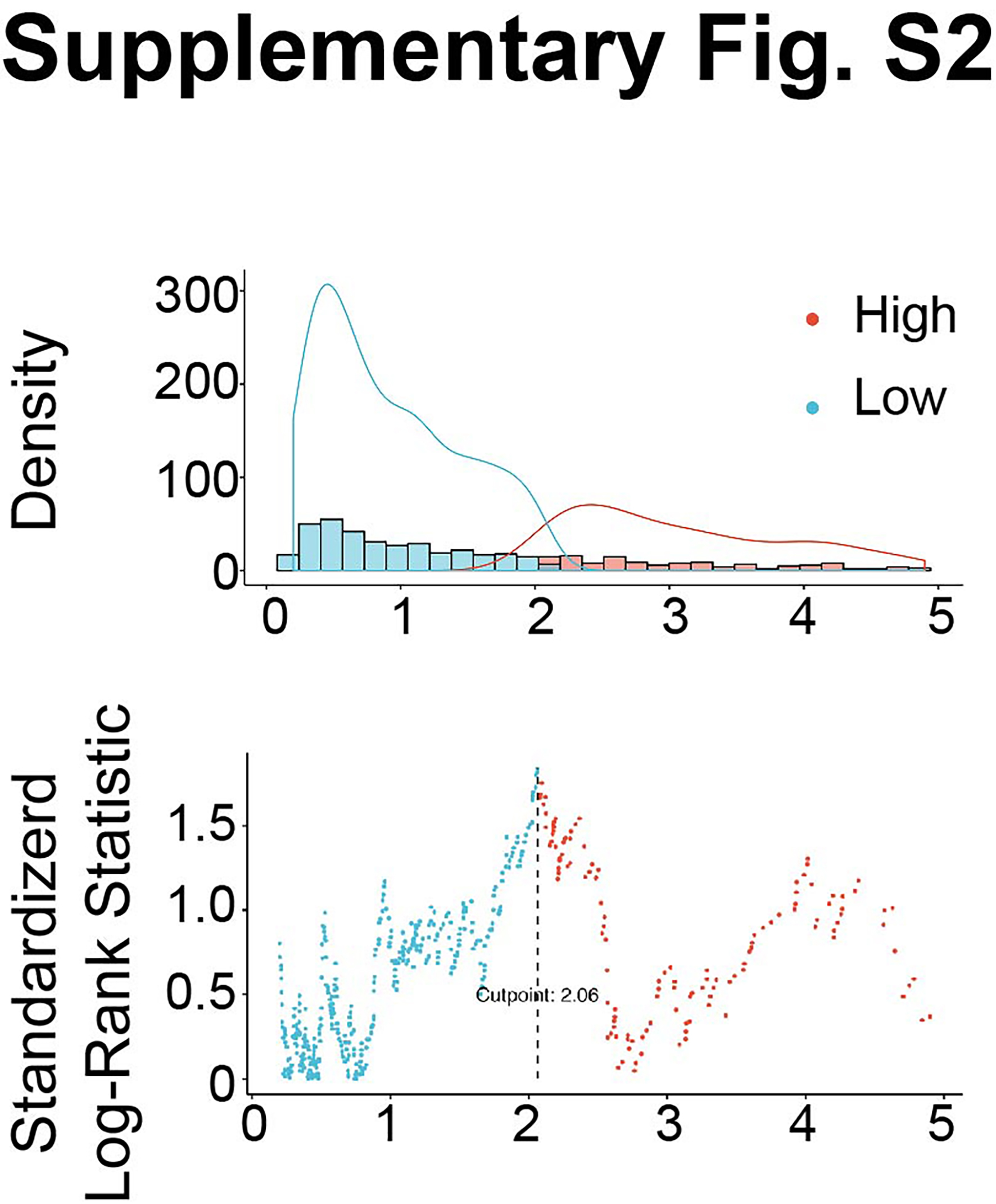

Supplement: Supplementary Figure 2 — The plot shows the optimal cutpoint for the stratification of TCGA CRC cohort into two groups, FAP-high and FAP-low, for overall survival analysis. [file Image_2.JPEG]

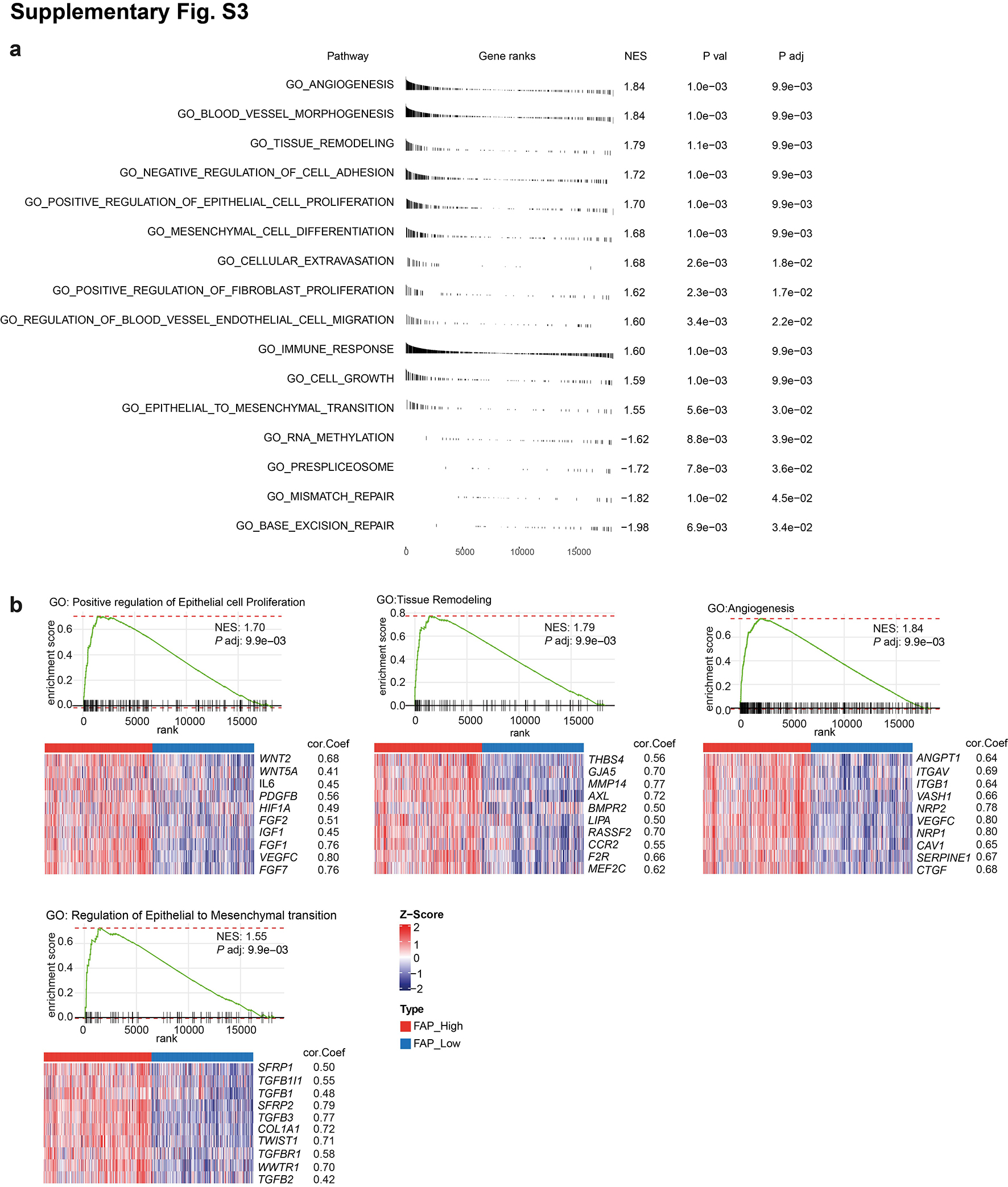

Supplement: Supplementary Figure 3 — (A) Gene Set Enrichment Analysis plot shows selected significantly enriched Gene Ontology gene sets from MSigDB database in FAP-high tumors in the TCGA CRC cohort. (B) Gene Set Enrichment Analysis plots of Gene Ontology molecular functions, where x-axis shows ranked list of genes (ranked by the p-values signed according to the direction of the differential expression analysis between FAP-high and FAP-low CRCs) and the vertical bars on the x-axis show the genes that belong to gene set. The y-axis shows the enrichment score of the gene set. Heatmaps below show selected genes in each GO process. Pearson correlation coefficients of the expression of the genes with FAP expression are shown to the right. NES: normalized enrichment score. P adj, p-value adjusted for multiple testing (i.e., FDR). [file Image_3.JPEG]

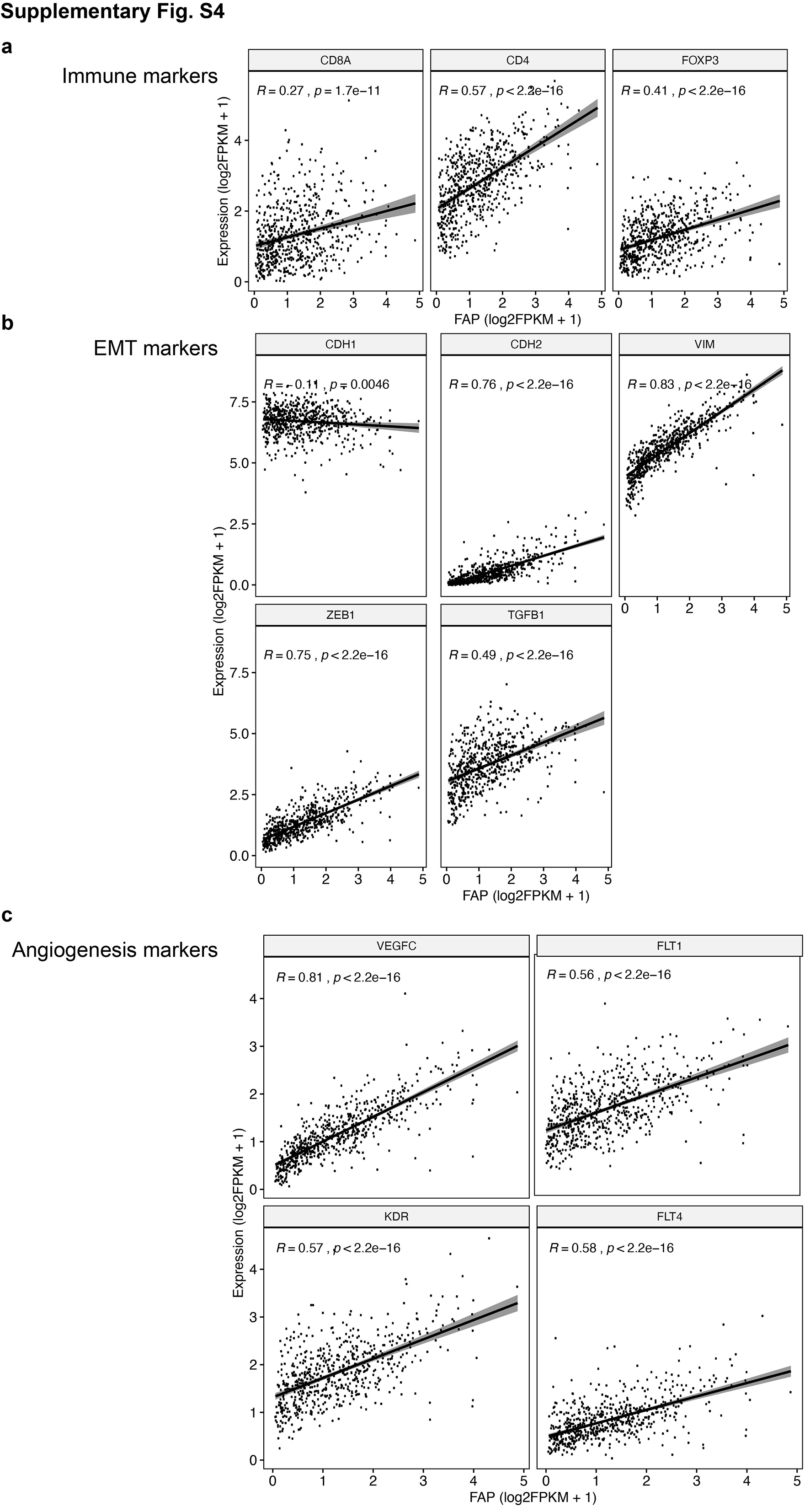

Supplement: Supplementary Figure 4 — Correlation of FAP expression with (A) immune markers; (B) epithelial to mesenchymal transition (EMT) markers and (C) angiogenesis markers. Statistical analyses were performed using Pearson correlation tests. [file Image_4.JPEG]

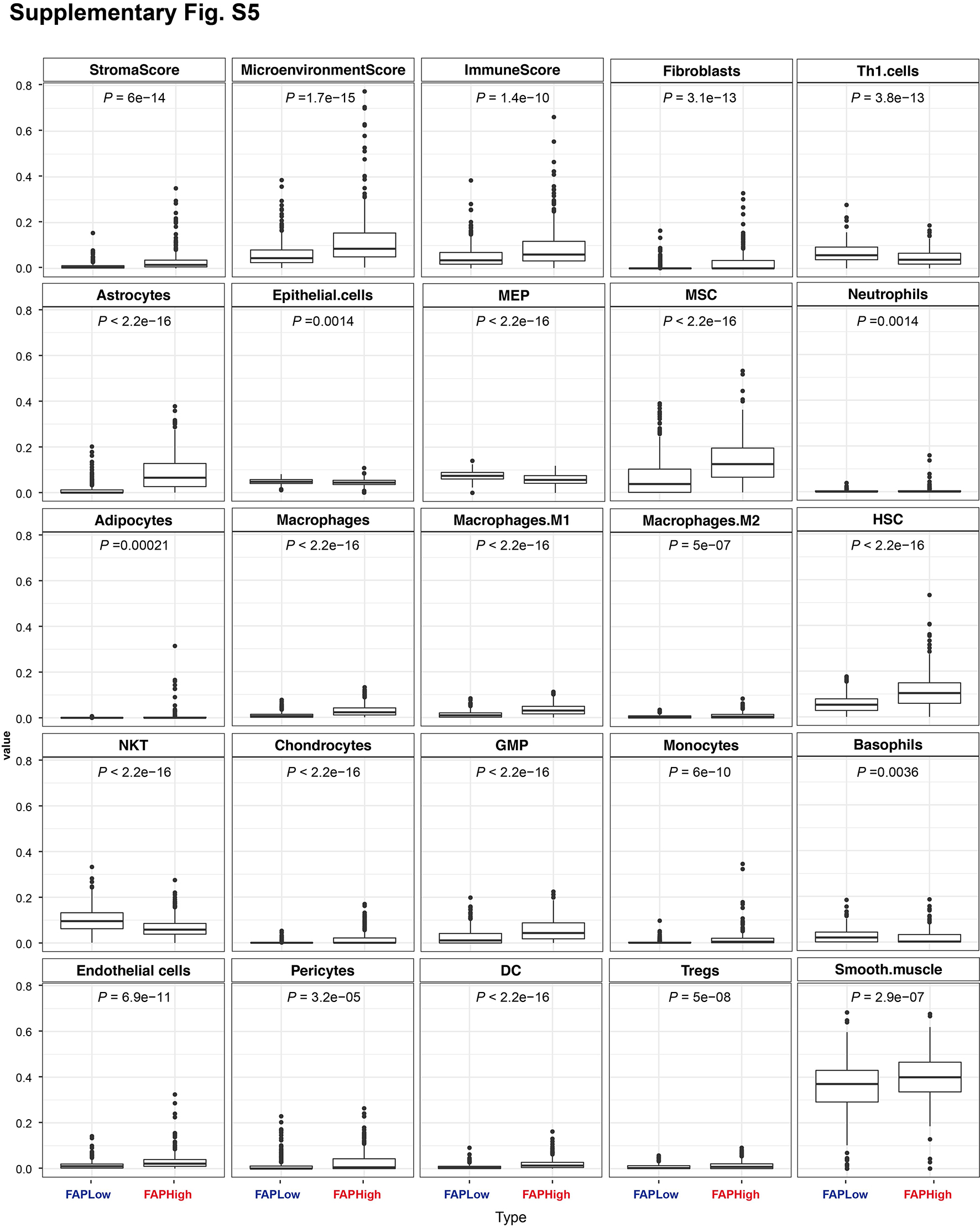

Supplement: Supplementary Figure 5 — Boxplots show the enrichment scores of each cell type between FAP-high and FAP-low groups in the TCGA CRC cohort, as defined by xCell. p-values were calculated using t-tests. [file Image_5.JPEG]
